# The relationship between serum 25-hydroxyvitamin D levels and the severity of COVID-19 disease and its mortality

**DOI:** 10.1038/s41598-021-97017-9

**Published:** 2021-09-02

**Authors:** Maryam Vasheghani, Nasrin Jannati, Parvaneh Baghaei, Mitra Rezaei, Roqayeh Aliyari, Majid Marjani

**Affiliations:** 1 Chronic Respiratory Diseases Research Center, National Research Institute of Tuberculosis and Lung Diseases (NRITLD), Shahid Beheshti University of Medical Sciences, Tehran, Iran; 2 Internal Medicine Department, School of Medicine, Shahid Beheshti University of Medical Sciences, Tehran, Iran; 3 Clinical Tuberculosis and Epidemiology Research Center, National Research Institute of Tuberculosis and Lung Disease (NRITLD), Shahid Beheshti University of Medical Sciences, Tehran, Iran; 4 Virology Research Center, National Research Institute of Tuberculosis and Lung Diseases, Masih Daneshvari Hospital, Shahid Beheshti University of Medical Sciences, Darabad, Niavaran, 196944413 Tehran, Iran; 5 Ophthalmic Epidemiology Research Center, Shahroud University of Medical Sciences, Shahroud, Iran; 6 Clinical Tuberculosis and Epidemiology Research Center, National Research Institute of Tuberculosis and Lung Diseases (NRITLD), Masih Daneshvari Hospital, Shahid Beheshti University of Medical Sciences, Tehran, Iran

**Keywords:** Biochemistry, Immunology, Diseases, Endocrinology, Medical research, Risk factors

## Abstract

Supplemental vitamin D can reduce the risk and mortality of viral pneumonia. The relationship between 25 hydroxyvitamin D [25(OH)D] levels and the severity and mortality of Coronavirus disease 2019 (COVID-19) was evaluated. In this cross-sectional study, the admitted patients with COVID-19 were categorized as mild, moderate, severe, and critical based on clinical and radiologic characteristics. Calcium, phosphorus, albumin, creatinine, and serum 25(OH)D were measured and their correlation with the severity of disease and mortality were analyzed. During 2 months, 508 patients (442 patients in general wards and 66 patients in the intensive care unit (ICU)) were included. The participants were 56 ± 17 years old (52% male, 37% with comorbidity). Concerning severity, 13%, 42%, 36%, and 9% had mild, moderate, severe, and critical diseases, respectively. The mortality rate was 10.8%. Admission to ICU, severity of disease and mortality decreased significantly across quartiles of 25(OH)D. According to multivariate logistic regression analysis, disease mortality had a positive correlation with age and had a negative correlation with the serum level of 25(OH)D, calcium, and albumin. In hospitalized patients with COVID-19, low 25(OH)D was associated with severe disease and increased ICU admission and mortality rate.

## Introduction

In late 2019, a new coronavirus was identified as a cause of a cluster of pneumonia cases in China which is named COVID-19 disease^1^. Currently, COVID-19 is pandemic^2^. In Iran, 612,772 people as the definitive cases of COVID-19 have been reported by November 10, 2020, and 34,864 people have died^3^. Manifestations of the COVID-19 range from asymptomatic carriers to acute respiratory failure and death^4^. Complications include acute respiratory failure, cytokine release syndrome, increased coagulation factors, and multi-organ damage which are associated with poor prognosis^5,6^. The overall mortality rate until November 18, 2020, is about 2.4% (1,333,742 deaths between 55,326,907 patients)^7^. Old age, cardiovascular disease, diabetes, high blood pressure, chronic lung disease, cancer, chronic kidney disease, people with defective or suppressed immune systems, obesity, and chronic liver disease have been identified as risk factors for severe disease or mortality^4,6,8,9^. There is currently no specific treatment against COVID-19 disease^10^. Currently, the most important way to deal with this disease is prevention and control of the conditions that are considered as a risk factor for the more severe course, and complications.

There is evidence from influenza A and severe acute respiratory syndrome (SARS) epidemics suggesting a role for vitamin D in these diseases^11^. Previous studies have suggested an association between vitamin D deficiency and an increased chance of developing bacterial and/or viral pneumonia due to viruses such as SARS, MERS, and Influenza A. COVID-19 disease is more prevalent and severe in winter and is more common in people who are more likely to be deficient in vitamin D, such as people with obesity and diabetes mellitus, and people who live in higher latitudes^12,13^.

About one-half of Iranian people have vitamin D deficiency (25 Hydroxyvitamin D < 20 ng/dl) and the burden of COVID-19 is catastrophically rising in Iran with a mortality rate of about 6%^14^. So, this study investigates the relationship between 25 Hydroxyvitamin D [25(OH)D] levels and the severity and mortality of COVID-19 to plan for improving patients care and reducing morbidity and mortality by appropriate treatment protocols or even planning for primary prevention in the next studies.

## Material and methods

This cross-sectional study was at Masih Daneshvari Hospital (tertiary center for lung disease and tuberculosis and nowadays, COVID-19), Tehran, Iran. Sampling was done by a simple sampling technique of available cases. Therefore, all patients who were hospitalized from April 1, 2020 to June 31, 2020, due to COVID-19, and their serum 25(OH)D levels were checked were included in the study according to the inclusion and exclusion criteria. Patients with COVID-19 who were hospitalized for at least 24 h were recruited and Cases with an uncertain diagnosis of COVID-19, pregnant women, and those without measurement of serum vitamin D levels were excluded. Informed consent is obtained for participation from participant and their medical history and clinical examination were obtained and recorded in the questionnaire. Patient height and weight were measured with a Seca stadiometer and digital scale. Diagnosis of COVID-19 was made based on reverse transcriptase-polymerase chain reaction (RT-PCR) assay for SARS-CoV-2 from nasopharyngeal or oropharyngeal sampling or a set of symptoms and chest CT scan findings consistent with viral infections by an infectious disease specialist. The RT-PCR test result for SARS-COV-2 is reported as positive or negative. The patients were categorized based on disease severity as mild, moderate, severe, and critical based on clinical symptoms, O_2_ saturation, and chest imaging (see definition part). Three milliliters of blood were taken from any patient for biochemical tests and a serum level of 25(OH)D. Measurement of calcium, phosphorus, albumin, creatinine was performed by auto analyzer due photometric method (diagnostic kit of Pars Azmoon Company, Tehran, Iran), and serum level of 25(OH)D was measured by chemiluminescent immunoassay (CLIA) (Atellica IM Vitamin D Total Assay, Siemens Company kit, Munich, Germany). The kit used to measure 25 hydroxyvitamin D in this study is standardized according to Vitamin D Standardization-Certification Program (VDSCP). The assay range of this kit is 4.2–150 ng/ml with the limit of quantitation of 3 ng/ml and calibration interval every 28 days. This kit has a 1% cross-reactivity to 3-epi. The results of total CV and mean bias in different levels of 25(OH)D are less than 5%^15^. References value in our laboratory are as definition of vitamin D status (see below).

The clinical and paraclinical parameters related to the severity of the disease were recorded in standard hospital forms for all patients based on the medical history and physical examination by physicians who work in emergency and general wards or ICU. The results of chest imaging have been reported by the radiologists according to the Fleischner Society criteria for interpreting chest CT scan findings consistent with the diagnosis of viral pneumonia (including COVID-19 disease)^16^. The outcome of the disease was classified as partial recovery, complete recovery, and death during hospitalization. Serum levels of 25(OH)D were not available at the time of recording clinical information such as disease severity and mortality. So, the disease severity and mortality were done blinded to lab data.

### Definitions

*Vitamin D status* has categorized as below^17–20^:

- Severe vitamin D deficiency: 25(OH)D <10 ng/ml.
- Moderate vitamin D deficiency: 25(OH)D = 10–20 ng/ml.
- Mild vitamin D deficiency: 25(OH)D = 21–30 ng/ml.
- Vitamin D adequacy: 25(OH)D = 30–100 ng/ml.
- Upper level Vitamin D: 25(OH)D > 100–149 ng/ml.

#### Vitamin D quartiles

25(OH)D is divided in quartiles. The cut-off points for 25(OH)D quartiles (25, 50, and 75) are 14, 24, and 37 ng/ml, respectively.

#### Vitamin D supplementation

Those who received at least 50,000 units of vitamin D in the past month.

#### Definitive COVID-19 infection

A patient who has at least one PCR test of his or her respiratory sampling positive for the SARS-CoV-2 virus^21^.

#### The severity of COVID-19 disease

According to the guidelines of the World Health Organization based on the patient’s respiratory status at the time of blood sampling, oxygen in the blood at rest and room air, as well as the respiratory rate were divided into four groups: mild, moderate, severe, and Critical^21^.

Mild pulmonary involvement:

- O_2_ sat at rest > 93% in room air and respiratory rate < 30 and normal lung CT.

Moderate pulmonary involvement:

- O_2_ sat at rest > 93% in room air and respiratory rate < 30 and involvement in lung CT.

Severe pulmonary involvement:

- O_2_ sat at rest < 93% in room air and/or respiratory rate > 30 and involvement in lung CT.

Critical pulmonary involvement:

I. Need nasal high flow oxygen therapy.
II. Requires intubation and mechanical ventilation.
III. Acute respiratory distress syndrome (ARDS).

#### Outcome COVID-19 disease

##### Partial recovery

Need O_2_ therapy after discharge from the hospital or unable to live at home alone and needs to help with self-care.

#### Body mass index (BMI)

BMI is obtained by dividing weight in kilograms by height squared in meters^22^.

### Statistical analysis

All data were entered into SPSS statistical software (IBM Corp. Released 2013. IBM SPSS Statistics for Windows, Version 22.0. Armonk, NY: IBM Corp.) and analyzed. A P-value less than 0.05 was considered statistically significant. Participants were divided into four groups based on the severity of the disease. For some quantitative variables when did not have a normal distribution in the Kolmogorov–Smirnov normality test, the nonparametric tests have been applied. The relation between these variables with disease outcome (complete recovery or partial recovery and death) was compared by Mann–Whitney Test. Chi-Square tests were used to examine the relationship between disease severity and outcome with disciplinary variables such as gender, comorbidities (diabetes, hypertension and ischemic heart disease, immune suppression disease), or history of glucocorticoid or vitamin D supplement usage.

The serum level of 25(OH)D is divided into quartiles. The relationships of demographic (age, sex) and medical parameters (admision ward and history of DM, IHD, glucocorticoid use, and vitamin D supplement use) of participants, and disease severity and outcome were compared across quartiles of serum 25(OH)D by analysis of covariance, then the Bonferroni post hoc test was done for specific between-group comparisons.

The association between the serum level of 25(OH)D as a continuous independent variable and in-hospital mortality were determined using binary logistic regression analysis with adjustment for age, sex, BMI, creatinine, calcium, phosphor, albumin, and history of comorbidities (diabetes mellitus, hypertension, and ischemic heart disease). Data are presented as β regression, 95% confidence interval, and odds ratio (OR) of 25 (OH)D for in-hospital mortality vs. recovery.

### Ethics approval and consent to participate

In this research, the ethical principles of research have been observed according to the Helsinki Convention. Written informed consent has been obtained for participation from the participant. All data is confidential. The data will be printed in groups without mentioning the names and personal details of the participants. The proposal of this plan was approved by the Ethics Committee in Biomedical Research of the National Research Institute of Tuberculosis and Lung Diseases—Shahid Beheshti University of Medical Sciences with the approval ID: IR.SBMU.NRITLD.REC.1399.132 on May 26, 2020.

### Consent for publication

The authors wish to submit an original research article entitled “The relationship between serum 25-hydroxyvitamin D levels and the severity of COVID-19 disease and its mortality” for consideration by *Scientific Reports*. We confirm that this work is original and has not been published elsewhere, nor is it currently under consideration for publication elsewhere.

## Results

During the study period, 556 patients were admitted, 482 cases in general wards, and 72 individuals in Intensive Care Units (ICU). Due to the death during the first 24 h of admission, failure to send the patient’s blood sample for the requested tests, or failure to perform a 25(OH)D test and exclusion criteria, only available data related to 442 patients admitted to the general wards and 66 patients admitted to the ICU were analyzed. The mean age of participants was 56 ± 17 years (range from 14 to 95 years) and 52% were male. According to the past medical history from the patients, 190 (37.4%) of patients had comorbidities (diabetes mellitus, ischemic heart diseases, hypertension, etc.). Concerning disease severity, 13%, 42%, 36%, and 9% had mild, moderate, severe, and critical diseases, respectively. In this study, 44% of patients needed advanced respiratory care, and 13% needed to be admitted to the ICU. The in-hospital mortality rate was 10.8%. People with critical illness had higher age, lower serum levels of 25(OH)D, calcium, albumin, and fewer vitamin D supplements usage, higher mortality rather than those with mild disease. The relationship between demographic and medical parameters with the severity of COVID-19 disease is listed in Table 1. Based on the Bonferroni post hoc test, the mean age among patients with mild disease was significantly different from the mean age of patients in other groups (P-values < 0.003). The mean level of 25(OH)D among patients with the critical status was significantly different from other groups (P-values < 0.04). The mean phosphorus levels in patients with mild and moderate status significant differences (P-value = 0.04) were observed. There was a significant difference between the mean calcium in critical disease with moderate disease (P-values < 0.0001) and mild disease (P-values < 0.0001). Also, the mean calcium levels in patients with severe disease and patients with mild disease (P-value = 0.02) were different.

**Table 1:** Relationship between demographic and medical parameters with the severity of COVID-19 disease.

Variables	Total (n = 508)	Disease severity				P-value
		Mild (n = 68)	Moderate (n = 217)	Severe (n = 157)	Critical (n = 66)	
Age^a^ (year)	56 ± 17	48 ± 17	56 ± 17	56 ± 16	63 ± 15	0.001
Sex; male, n (%)	264 (52)	42 (62)	103 (47)	81 (52)	38 (58)	0.15
BMI^a^ (kg/m^2^)	27 ± 5	27 ± 4	27 ± 5	27 ± 5	27 ± 5	0.95
**Medical history; yes, n (%)**						
DM	116 (23)	13(19)	43 (20)	41 (26)	19 (29)	0.27
HTN	35 (7)	1 (1)	16 (7)	13 (8)	5 (8)	0.29
IHD	72 (14)	7 (10)	30 (14)	23 (15)	12 (18)	0.62
Immune system suppression	31 (6)	2 (3)	11 (5)	15 (8)	3 (6)	0.38
Glucocorticoid use	27 (5.3)	2 (3)	15 (7)	8 (5)	2 (3)	0.48
Vitamin D supplement use	88 (17)	14 (21)	42 (19)	31 (20)	1 (1)	0.004
**COVID-19 disease outcome, n (%)**						0.001
Death	55 (10.8)	0 (0)	3 (1)	31 (17)	21 (46)	
Partial recovery	253 (50)	12 (18)	111 (52)	111 (62)	19 (41)	
Complete recovery	200 (39)	55 (82)	101 (47)	38 (21)	6 (13)	
25 OH vitamin D^a^ (ng/ml)	28.6 ± 21.6	31.8 ± 25.7	30.4 ± 22.4	28.2 ± 20.8	20.4 ± 16.3	0.001
Creatinine^a^ (mg/dl)	1.4 ± 1.2	1.5 ± 1.9	1.3 ± 1.0	1.4 ± 1.1	1.6 ± 1.0	0.28
Calcium^a^ (mg/dl)	8.8 ± 0.7	9.0 ± 0.8	8.8 ± 0.6	8.7 ± 0.6	8.5 ± 0.8	0.001
Phosphor^a^ (mg/dl)	3.3 ± 1.06	3.6 ± 0.8	3.2 ± 0.9	3.1 ± 0.8	3.4 ± 1.8	0.02
Albumin^a^ (g/dl)	3.4 ± 0.6	3.9 ± 0.5	3.5 ± 0.5	3.5 ± 0.8	3.0 ± 0.4	0.001

*DM* diabetes mellitus, *HTN* hypertension, *IHD* ischemic heart disease, *BMI* body mass index.

^a^Mean ± SD.

**Figure 1:**
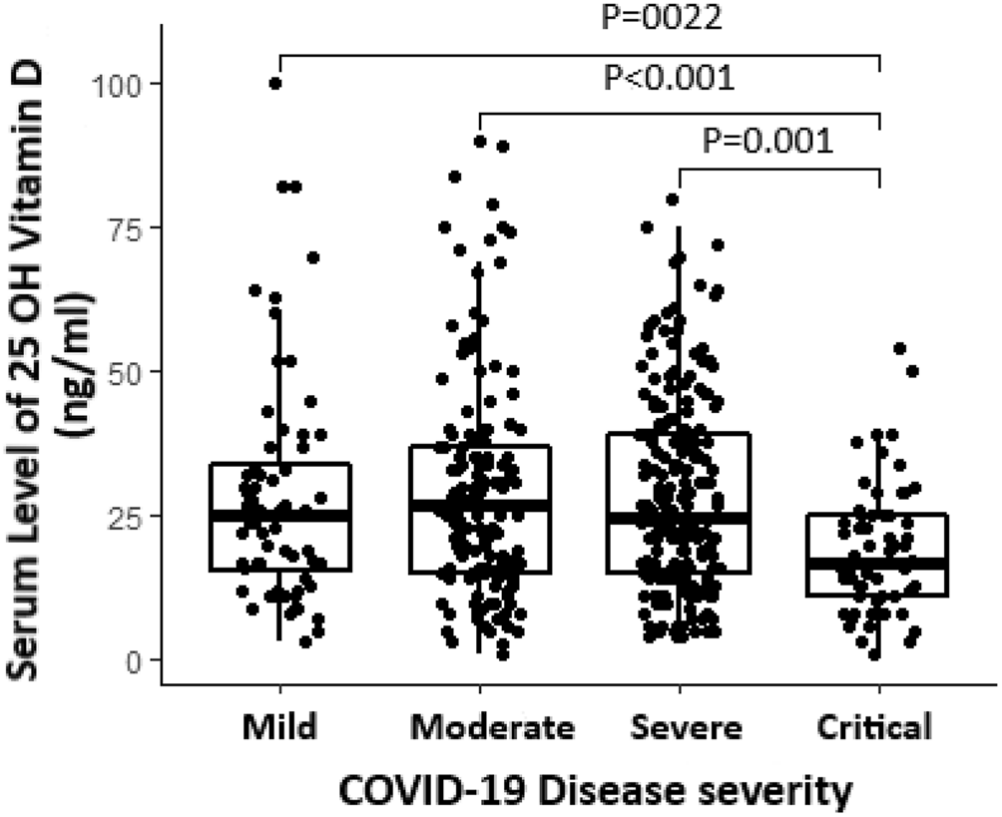
Comparative diagram of median and interquartile of serum 25 hydroxyvitamin D level in different intensities of COVID-19 disease.

Figure 1 is a box plot showing median and interquartile range of 25(OH)D and different levels of disease severity. The comparisons between median serum vitamin D levels based on severity of disease examined by Mann–Whitney test due to the abnormal distribution of serum 25(OH)D levels. The P-values in Fig. 1 represent the statistically differences between groups. Vitamin D deficiency (25(OH)D < 30 ng/ml) was more common in women than men (69% vs. 56%, P = 0.003) and patients under ICU care than patients admitted in other wards (80% vs. 61%, P = 0.01). The prevalence of vitamin D deficiency and critical illness was lower in people taking vitamin D supplements (P = 0.001 for both). The prevalence of vitamin D deficiency was not different between patients with a history of comorbidities and those without it.

Table 2 summarized the patient’s demographic and medical characteristics based on the outcome. The mortality is directly related to the patient’s age and the severity of the disease. The mortality of people older than 60 years was five times higher than that of people 30 or younger. Mortality is inversely related to serum levels of 25(OH)D, calcium, albumin, and renal function.

**Table 2:** Demographic and medical characteristics of 508 cases of COVID-19 disease categorized by outcome.

Variables	COVID-19 disease outcome			P-value
	Death n = 55	Partial recovery n = 253	Complete recovery n = 200	
Age^a^	65 ± 15	58 ± 17	51 ± 16	0.001
Sex ; male, n (%)	25 (45.5)	118 (46.6)	101 (51)	0.66
BMI^a^ (kg/m^2^)	26 ± 5	27 ± 5	27 ± 5	0.61
**PMH; yes, n (%)**				
DM	13 (23.6)	64 (25.3)	39 (20)	0.34
HTN	4 (7.3)	22 (8.7)	9 (5)	0.21
IHD	8 (14)	44 (17)	20 (10)	0.08
Immune system suppression	6 (10.9)	16 (6.3)	9 (5)	0.21
Glucocorticoid use	4 (7.3)	12 (4.7)	11 (6)	0.74
Vitamin D supplement use	7 (12.7)	47 (18.6)	34 (17)	0.58
**COVID-19 disease severity, n (%)**				0.001
Mild	0 (0)	12 (5)	55 (28)	
Moderate	3 (6)	111 (44)	113 (50)	
Severe	31 (56)	111 (44)	38 (19)	
Critical	21 (38)	19 (7)	6 (3)	
25OH vitamin D^a^^,^* (ng/ml)	24 ± 19	30 ± 22	30 ± 22	0.047
Creatinine^a^ (mg/dl)	2 ± 1	1 ± 1	1 ± 1	0.007
Calcium^a^ (mg/dl)	9 ± 1	9 ± 1	9 ± 1	0.001
Phosphor^a^ (mg/dl)	3 ± 2	3 ± 1	3 ± 1	0.25
Albumin^a^ (g/dl)	3 ± 1	4 ± 1	4 ± 1	0.001

*DM* diabetes mellitus, *HTN* hypertension, *IHD* ischemic heart disease, *BMI* body mass index.

^a^Mean ± SD.

*The P-value has been calculated after recategorized disease outcome to recovery and death groups.

The cut-off points for 25(OH)D quartiles (25, 50, and 75) are 14, 24, and 37 ng/ml, respectively. The ICU admission rate, and severity of disease and mortality decreased significantly across quartiles of 25(OH)D. The relationships of demographic and medical parameters of participants, and disease severity and outcome across quartiles of serum 25(OH)D is shown in Table 3.

**Table 3:** The relationships of demographic and medical parameters across quartiles of serum 25(OH) D.

	Serum 25(OH)D quartiles				P-value
	Q1	Q2	Q3	Q4	
**Admision ward**^**a**^					
Non ICU	95 (78)	105 (85)	106 (90)	114 (95)	< 0.0001*
ICU	26 (22)	19 (15)	12 (10)	6 (5)	
**Sex**^**a**^					
Male	69 (57)	69 (56)	65 (55)	46 (38)	0.005*
Female	52 (43)	55 (44)	53 (45)	74 (62)	
**DM**^**a**^					
No	95 (78)	97 (78)	91 (77)	92(77)	0.69
Yes	26 (22)	27 (22)	27 (23)	28 (23)	
**IHD**					
No	103 (85)	108 (87)	102 (86)	103 (86)	0.92*
Yes	18 (15)	16 (13)	16 (14)	17 (14)	
**Glucocorticoid use**^**a**^					
No	118 (97)	114 (92)	113 (96)	114 (95)	0.68*
Yes	3 (3)	10 (8)	5 (4)	6 (5)	
**Vitamin D supplement use**^**a**^					
No	114 (94)	104 (84)	95 (80)	88 (77)	< 0.0001*
Yes	7 (6)	20 (16)	23 (20)	32 (23)	
**COVID-19 disease severity**^**a**^					
Mild	16 (13)	16 (13)	18 (15)	15 (13)	0.01
Moderate	48 (40)	54 (44)	43 (37)	62 (52)	
Severe	31 (26)	35 (28)	45 (38)	36 (30)	
Critical	26 (21)	19 (15)	12 (10)	6 (5)	
**COVID-19 disease outcome**^**a**^					
Death	17 (14)	14 (11)	13 (11)	6 (5)	0.03*
Recovery	104 (86)	110 (89)	104 (89)	113 (95)	
Age (year)^b^	54 ± 18	55 ± 17	56 ± 17	59 ± 16	0.17
BMI^b^	27 ± 4	26 ± 5	28 ± 5	28 ± 5	0.09
Creatinin (mg/dl)^b^	2 ± 1	2 ± 1	2 ± 2	1 ± 0	0.28
Cacium (mg/dl)^b^	9 ± 1	9 ± 1	9 ± 1	9 ± 1	0.44
Phosphor (mg/dl)^b^	3 ± 2	3 ± 1	3 ± 1	3 ± 1	0.50
Albumin (g/dl)^b^	3 ± 1	3 ± 1	4 ± 1	4 ± 1	0.05

*DM* diabetes mellitus, *HTN* hypertension, *IHD* ischemic heart disease, *BMI* body mass index.

^a^n (%).

^b^Mean ± SD.

*Cochran-Armitage test for trend.

In the multivariate regression analysis, in-hospital mortality increases 4% by age, and decrease 3%, 47% and 79% by serum levels of 25(OH)D, calcium, and albumin, respectively. Details were showed in Table 4.

**Table 4:** Multivariate odds ratio and 95% confidence for association between the serum level of 25 hydroxyvitamin D and COVID-19 disease mortality.

Independent variable	Multivariate analysis			
	Odds ratio	95% CI for EXP(B)		P value
		Lower	Upper	
Age	1.04	1.02	1.06	< 0.001
Sex (male)	0.89	0.51	1.56	0.69
BMI	0.97	0.92	1.03	0.35
25 OH vitamin D	0.97	0.96	0.99	0.04
Diabetes mellitus	1.05	0.54	2.02	0.89
Hypertension	0.79	0.26	2.42	0.68
Ischemic heart disease	0.68	0.28	1.62	0.38
Creatinine	1.08	0.90	1.30	0.39
Calcium	0.53	0.36	0.80	0.002
Phosphor	1.13	0.90	1.43	0.29
Albumin	0.21	0.11	0.38	< 0.001

*COVID-19* coronavirus 2019 disease, *CI* confidence interval, *BMI* body mass index.

Mortality vs. recovery.

After adjustment for sex, BMI, past history of diabetes mellitus, hypertension, ischemic heart disease, creatinine, phosphor, albumin.

Odds ratio change in mortality per standard deviation increase in scale independent variable. Each odds ratio result is adjusted for each of the other independent variables in the table.

## Discussion

In this cross-sectional study, 556 patients were studied. There was a negative correlation between disease severity and history of vitamin D supplementation, serum levels of 25(OH)D, calcium, phosphorus, and albumin. Also, there was a negative correlation between in-hospital mortality and serum levels of 25(OH)D and calcium. The rate of admission to ICU, and disease severity and mortality rate decreased significantly across quartiles of 25(OH)D.

Age was both a risk factor for COVID-19 disease and its severity in this study. This finding is consistent with the findings of previous studies. For every 5 years of increase in patient age in the USA, the rate of hospitalization and mortality increase by 34% and 10–18%, respectively^23,24^. The imbalance of the immune system and comorbidities intensifies the severity of the disease and consequently increases the mortality rate^25^.

In this study, there was no significant difference between gender and disease severity, and outcome. In a review article, the male-to-female ratio in patients admitted for COVID-19 was similar in three studies in France, Spain, and Switzerland. Male sex hormones, concurrent diseases, behavioral differences, and more exposure of men to pathogens may play a role in these differences^26^.

There was no relationship between comorbidities with the severity and outcome of COVID-19 disease in this study. Bajgain etal reported that there is no clear association between these comorbidities and mortality rate^27^. But, these factors have been associated with increased severity and mortality of COVID-19 disease in some other studies^28–30^. This discrepancy may be due to different methods of study and participant demographic characteristics such as age and the male-to-female ratio. In our study, the diagnosis of comorbidities was based on medical history. We didn’t have online access to the patient's previous medical records.

In this study, the number of patients admitted to ICU is 3.8 times and the in-hospital mortality rate is twice the globally national death rate in Iran^31,32^. This study was performed at the time of the first peak of the disease in Iran. At that time, the number of PCR tests performed to diagnose COVID-19 was low. Our hospital is a tertiary and referral center for respiratory diseases and COVID-19. Therefore, more serious patients refer to this center and one-fifth of our patients had over 70 years old. Of course, the in-hospital mortality rate in this study is lower than in other centers. About 32–40% of hospitalized patients need to be admitted to the ICU and their mortality rate is about 15–39% in other studies^33,34^. Experienced and well-trained personnel to care for patients with respiratory disorders and infectious diseases and access to adequate facilities for non-invasive and invasive ventilation can justify these results.

In the present study, 40% percent of patients had 25(OH)D less than 20 ng/ml. Contrary to expectations, although Iran is a sunny country, vitamin D deficiency is common in all age groups. The reason is the lack of intake through food, reduced synthesis of this vitamin in the skin, and the type of clothing, and lack of widespread supplementation, which is common in some countries^36–37^.

In the present study, the patients with low blood levels of calcium, phosphorus, and albumin had more severe diseases, poor outcomes, and mortality. Similar results were found in other published articles. In one study, among patients with COVID-19, serum calcium levels were 0.8 mg/dl lower than in other patients^38^. Patients who died of COVID-19 had a serum albumin level of 4.6 g/l lower than those who survived^39^. This relationship can be explained in several aspects. First, each of these nutrients have a special role in the function of the immune system and various parts of the body. Second, their deficiency of is an indirect index of the patient’s nutritional status or concomitant conditions such as obesity, renal and liver failure, and diabetes. Third, hypocalcemia due to hypoalbuminemia may affect these results and it is better to correct the amount of serum calcium according to albumin level.

There was a negative correlation between disease severity and serum levels of 25(OH)D in this study. The mean level of 25(OH)D in patients admitted to the ICU was 11 ng/ml less than other groups. This finding has been shown in other studies. In the acute critically ill patients with COVID-19, the mean level of vitamin D was lower (14 vs. 28 ng/ml), and the prevalence of vitamin D deficiency (25(OH)D < 20 ng/dl) was higher than asymptomatic patients(96 vs. 33%). In patients with COVID-19 and concomitant vitamin D deficiency, and the mortality rate was 7 times (21 vs. 3%) than those with sufficient vitamin D^40^.

In a cohort study by Baktash et al.^41^, the median level of 25(OH)D was lower inpatients with COVID-19 than healthy controls. This difference in 25(OH)D between the two groups was much greater than their study (62 ng/ml). Of course, they have compared patients with healthy people and their participants were older. Unlike previous studies, Jevalikar^42^ and Panagiotou^43^ did not find any relationship between 25(OH)D level and clinical parameters, inflammatory markers, or mortality rate in COVID-19 patients. The different results reported in these studies may be due to significant differences in the characteristics of participants and the presence of comorbidities. However, Panagiotou also found vitamin D deficiency was more common in patients admitted to the ICU. They treated patients with vitamin D supplements immediately after the diagnosis of vitamin D deficiency, which may have affected the course of the disease.

Vitamin D supplementation was associated with reduced disease severity and mortality in this study. In a few clinical trials, vitamin D administration has reduced the risk of COVID-19 disease, its morbidity, and mortality^44–46^. In a retrospective study, people with a history of a low mean level of 25(OH)D were more likely to develop COVID-19 disease than those with a higher level of 25(OH)D (11 vs. 25 ng/ml, respectively)^47^. Although in another study, a high dose of vitamin D has did not affect on the course of COVID-19 disease, and comments on this subject need further investigations^48^.

In this study, age was directly related to mortality, but disease severity, serum levels of 25-hydroxyvitamin D, creatinine, calcium, and albumin were inversely related to mortality due to COVID-19 disease.

There are pieces of evidence to suggest the link between 25(OH)D levels and COVID-19 disease. Vitamin D deficiency may increase the risk of COVID-19 disease and its severity and mortality. In a cohort study of 185 patients with COVID-19 within 66 days, 50% were admitted, 28 required mechanical ventilation, and 16 died. When adjusted for age, gender, and comorbidities, 25(OH)D < 12 ng/ml was associated with a higher risk of death (HR 14.73, 95% CI 4.16–52.19, P < 0.001)^49^. In a retrospective cohort study, the relative risk and predicted rate of COVID-19 disease was higher in patients with vitamin D deficiency than in those with sufficient vitamin D [1.77 and 1.8 times, respectively)^50^. Vitamin D deficiency may also increase the risk of COVID-19 and disease severity and mortality. In a meta-analysis, a serum level of 25(OH)D < 30 ng/ml increased the rate of hospital admissions and the mortality rate from COVID-19 (OR 1.82 for both)^51^. The mortality rates 10 days after admission in patients with COVID-19 disease and severe vitamin D deficiency [25(OH)D < 10] ng/ml was 10 times the other group (25(OH)D ≥ 10 ng/ml)^52^.

Some studies did not find any relationship between the serum level of 25-hydroxyvitamin D and the severity of COVID-19 disease and mortality. In a retrospective population-based study in Brazil, 14,692 people who recently measured serum levels of 25(OH)D and had RT-PCR test for COVID-19 were studied^53^. There was no significant difference between PCR positive and PCR negative individuals in mean 25(OH)D levels and the prevalence of vitamin D deficiency.

In a retrospective cohort study on 347 patients who hospitalised due to COVID-19 disease in Italy, similar results were obtained^54^.

In severe COVID-19 disease, the immune system does not have the proper response to prevent the multiplying and progressing virus infection. Instead, cytokine storms occur due to the release of excessive inflammatory factors^55^. The relationship between Vitamin D and COVID-19 severity and outcome can be explained by different pathways and mechanisms, some of which we are mentioned here. Vitamin D has an antibacterial and antiviral property by regulating innate and adaptive cellular immunity, and physical barriers^56^. Vitamin D produces antimicrobial peptides (AMPs) such as cathelicidins and defensins by activating immune cells. A primary form of Cathelicidins (LL-37) inactivate viruses such as the Influenza A virus by destroying envelope proteins^57^. In the presence of a “cytokine storm” due to severe COVID-19 disease, inflammatory cytokines such as IL-6, IL-8, CRP, and ferritin are released without the control of the immune system. Inflammatory cytokines damage the integrity of the lungs by causing inflammation, leading to pneumonia, which in turn causes a vicious cycle^58^. IL-6 increases the severity of COVID-19 by rearranging the angiotensin-converting enzyme (ACE2) receptors and inducing macrophage cathepsin L. Cathepsin L of macrophage cleaves the S1 subunit of the coronavirus spike glycoprotein. This is essential for the coronavirus to enter human host cells, a fusion of the endosome membrane of a host cell for virus, and the release of viral RNA^59^. Vitamin D can modulate the immune system and reduce the production of pro-inflammatory markers. Vitamin D supplementation has reduced interleukin-6 levels in several clinical trials^60^. Vitamin D may reduce the risk of ARDS and mortality from COVID-19 by raising ACE2 levels. The SARS-COV-2 virus binds to the ACE2 receptor expressed on the surface of lung epithelial cells and causes over-accumulation of angiotensin II by ACE2 downregulation. In the in-vivo studies, vitamin D-binding protein has played a role in this interaction. Calcitriol, the active metabolite of vitamin D, increases ACE2 expression in the lungs in animal studies. Vitamin D replacement may reduce lung damage by increasing ACE2 expression and synthesis of α-1-antitrypsin by CD4+ T cells. α-1-Antitrypsin is critical for lung integrity and repair and is required for the further production of anti-inflammatory interleukins such as IL-10. Vitamin D improves endothelial dysfunction by reducing the oxidative stress of free oxygen radicals, TNF-alpha and interleukin-6 and suppressing the NF-κB pathway. Endothelial dysfunction causes vascular inflammation and increased blood coagulation, which is seen in severe COVID-19. Vitamin D reduces the lung damage due to COVID-19 by stimulating the proliferation and migration of alveolar epithelial cells type II and reducing their apoptosis. It also inhibits the mesenchymal transition of an epithelial cell induced by TGF-β. In COVID-19, the function of type II pneumocytes is impaired, and the surfactant concentration decreases at the alveolar surface, and the alveoli are collapsed. In some studies, 1α, 25 (OH) 2D have caused an increase in surfactant and maybe prevent lung alveoli collapse^62–63^. Studies have shown that acute inflammatory disease can decrease serum levels of 25(OH)D. These changes were temporary and short-lived and resolved within 24–48 h. There is a significant diurnal and seasonal fluctuation (20%) in the serum level of 25(OH)D in each person during the day and its maximum amount is observed in the middle of the day and the summer and autumn. Due to this daily fluctuation, the sampling time makes a significant error in assessing the serum level of 25(OH)D^65–67^. Therefore, the timing of blood sampling from patients from the onset of symptoms and disease or blood drawing time can change the results. Although all participants entered this study in May and June, and fasting sampling was performed in the morning of the first day after admission.

This study had some limitations. First, vitamin D-binding protein was not measured, and since the amount of this protein decreases in many severe diseases, the potential impact of this reduction must be considered. Second, vitamin D levels were measured once, and if measured several times in the course of the disease we might be able to better answer its relationship to the severity and outcome of the disease. Third, Vitamin D levels are measured during May and June. Vitamin D levels are lower during the winter and spring than the summer and autumn^68^. The timing of the study may affect the results obtained in this study. Fourth, This study is a single-center study and all participants are Caucasians who live in the urban area. As a result, it may have high internal validity and low external validity. In any case, a cross-sectional study with this style can not examine the causal relationship between variables and we did not intend to conclude the cause-and-effect relationship between vitamin D and the severity of COVID-19.

However, judgments based on these limited studies are not logical. Most of these studies are observational, single-center, retrospective and with inadequate sample size. There are limited human or financial resources and a lack of enough time due to the COVID-19 disease pandemic. So, acceleration in the research project design and implementation, data collection and analysis, and publishing research results also affect the quality of articles. We suggest multinational, multicenter, double-blind randomized clinical trials or cohort studies clarify the issue.

## Conclusion

This study evaluated the association between 25(OH)D levels and the severity and outcome of patients with COVID-19 admitted to the hospital. The disease was severe or critical in 44% of admitted cases and 13% of them admitted to ICU. The mortality rate was 10.8%. Old age, vitamin D deficiency, hypocalcemia, hypophosphatemia, hypoalbuminemia, and renal failure are associated with disease severity and admission to ICU. Old age, disease severity, low serum level of 25(OH)D, hypocalcemia, hypoalbuminemia, and renal failure were associated with a higher mortality rate.

## Data Availability

All authors have had online access to patients’ records and data during the study.
